# Integrative Transcriptome Analysis and WGCNA Uncover the Growth Regulatory Mechanisms in *Cephalopholis sonnerati*

**DOI:** 10.3390/ani16081128

**Published:** 2026-04-08

**Authors:** Ziyuan Wang, Yu Song, Runkai Sun, Zhenxia Sha, Yang Liu, Songlin Chen

**Affiliations:** 1 College of Life Science, Qingdao University, Qingdao 266071, China; wangziyuan2@qdu.edu.cn (Z.W.); sy302969975@163.com (Y.S.); shazhenxia@163.com (Z.S.); 2 State Key Laboratory of Mariculture Biobreeding and Sustainable Goods, Yellow Sea Fisheries Research Institute, Chinese Academy of Fishery Sciences, Qingdao 266071, China; rk4396000@gmail.com; 3 Laboratory for Marine Fisheries Science and Food Production Processes, Qingdao Marine Science and Technology Center, Qingdao 266237, China

**Keywords:** *Cephalopholis sonnerati*, transcriptomic analysis, weighted gene co-expression network analysis (WGCNA), pathway enrichment, hub genes

## Abstract

The tomato hind *(Cephalopholis sonnerati)* is a marine aquaculture fish species with high economic value. Elucidating the mechanisms underlying its growth regulation is crucial for the development of the aquaculture industry. To analyze the biological mechanisms underlying growth differences, individuals with extreme body sizes at 8 months of age from the same batch were selected in this study. A combined experiment of “body size × feeding status” was constructed, and transcriptome sequencing and weighted gene co-expression network analysis (WGCNA) were performed on brain and muscle tissues. The results showed that 2553 differentially expressed genes (DEGs) were identified between individuals with distinct body sizes, which were significantly enriched in growth regulation pathways such as PI3K–Akt, MAPK, and FoxO. Feeding differences affected 4480 genes, which were significantly enriched in signaling pathways including the insulin signaling pathway. WGCNA further identified co-expression modules (brown4, blue, coral1) significantly correlated with growth, as well as hub genes including *pik3r1* and *eif4ebp2*. Comprehensive analysis demonstrated that the growth regulation of *C. sonnerati* operates as a cascade network. Brain tissues perceive signals through neuroactive ligand–receptor interactions and integrate and transduce these signals via core pathways including Ras–MAPK and PI3K–Akt. Finally, growth processes are executed in muscle tissues by regulating glycogen metabolism, protein synthesis, and other processes, which are precisely regulated by terminal processes such as cellular senescence. Among them, *pik3r1* and *eif4ebp2*, as key molecular switches, play a central role in integrating upstream signals and precisely regulating downstream growth programs. This study preliminarily clarifies the molecular mechanism network of growth differences in *C. sonnerati*, providing a theoretical basis and candidate genes for the genetic improvement of its growth traits.

## Simple Summary

The *Cephalopholis sonnerati* is a valuable marine fish species widely farmed for food; however, the fundamental biological mechanisms governing its growth rate remain poorly understood. To address this knowledge gap, we examined 8-month-old tomato hind from the same cohort that exhibited markedly different body sizes (large vs. small individuals). Fish from each size group were randomly and equally assigned to two subgroups subjected to either a one-week starvation treatment or normal feeding conditions. Subsequently, brain and muscle tissues were collected for transcriptomic analysis. Through transcriptome sequencing and network analysis, we found that fish growth is regulated by an integrated system involving both tissues: the brain detects and initiates growth signals, while the muscle carries out growth processes such as protein synthesis and sugar metabolism. Our research uncovers the key biological network behind growth differences in tomato hind, and the findings provide essential information and key genetic targets to help farmers breed faster-growing tomato hind, supporting the sustainability and productivity of this important marine aquaculture industry.

## 1. Introduction

Groupers (*Epinephelinae*) are an important fish group in tropical and subtropical coral reef ecosystems worldwide. They are also among the most commercially valuable marine fish species in China, with an annual yield of 241,500 tons and a market value of approximately 42 billion US$ [1]. *Cephalopholis sonnerati* is a species of grouper belonging to the genus *Cephalopholis* [2]. As a benthic coral reef fish, *C. sonnerati* naturally inhabits tropical and subtropical regions of the Indo-Pacific [3]. *C. sonnerati* exhibits simultaneous hermaphroditism during growth. Females reach sexual maturity at a body length of 28 cm, whereas males reach sexual maturity at 34 cm. The maximum recorded body length of *C. sonnerati* is 57 cm [4]. Under artificial culture conditions, *C. sonnerati* shows significant individual differences in growth, which often lead to size differentiation within populations and, in severe cases, cannibalism. Therefore, elucidating the molecular mechanisms underlying growth differences in *C. sonnerati* is of great theoretical and practical importance for breeding improved varieties with uniform growth traits and achieving sustainable industrial development. To date, most studies on *C. sonnerati* have focused on the construction of a high-quality genome map, body coloration, immunity, embryonic development, cell line establishment, and sustainable development of fishery resources [1,5,6,7,8]. Research on the regulatory mechanisms of its growth and metabolism remains limited. Although the general framework of growth regulation in fish has been well established, the specific molecular mechanisms leading to growth differences in *C. sonnerati* remain unclear.

Growth is one of the most important economic traits in aquaculture breeding [9]. Investigating the growth characteristics of various fish species and elucidating the underlying mechanisms are key tasks for researchers. Numerous studies on growth mechanisms have been conducted in economically important marine fish species, including *Cynoglossus semilaevis*, *Larimichthys crocea*, and groupers. These studies have mainly focused on mapping growth-related loci [10,11,12,13], identifying associated markers and genes [14,15,16,17], and analyzing the expression patterns of growth-related genes [18,19,20]. However, growth is a typical quantitative trait controlled by multiple minor-effect polygenes and influenced by environmental factors [21]. Therefore, it is more practical to perform multi-level and in-depth analyses of differentially expressed genes (DEGs) in individual fish [22], in order to identify key growth-related genes and construct regulatory networks. Studying individuals from the same batch with similar genetic backgrounds but divergent growth rates is a commonly used strategy to investigate fish growth mechanisms. The core of this strategy is to identify growth-related loci and genes and enrich key pathways by comparing genomic and transcriptomic differences between fast-growing and slow-growing individuals, thereby revealing the molecular mechanisms underlying growth variation [23,24,25]. Nevertheless, growth is a complex process regulated by polygenic networks, and individual differences accumulate over long-term development. Subtle differences observed at a single time point are often difficult to detect under steady-state conditions. Starvation induces a series of adaptive physiological responses, including energy redistribution, reduced metabolic rate, and compensatory growth upon refeeding. Although these processes may influence the expression of feeding-related genes, they also provide a valuable framework for identifying key regulators involved in the coordinated control of growth and metabolism. To enhance the detection of such differences, a “starvation–feeding” nutritional intervention is often applied. This approach disrupts metabolic homeostasis by inducing fluctuations in nutritional status, thereby amplifying inherent differences in nutrient utilization and metabolic regulation among individuals [26,27].

In recent years, with the rapid development and reduced cost of high-throughput sequencing technology, transcriptome sequencing (RNA-seq) has become a widely used method for elucidating molecular mechanisms in biology [28]. In the field of hydrobiology, RNA–seq has been widely applied in studies on growth performance [29,30], immune response, and environmental adaptation of economic species such as fish and shellfish [31,32,33]. RNA–seq can comprehensively and rapidly generate gene expression profiles, providing a foundation for in-depth analysis of biological processes [34]. However, how to convert gene expression data into meaningful biological insights remains a major challenge in subsequent analysis. Weighted gene co-expression network analysis (WGCNA), as a powerful bioinformatics tool, is widely used to analyze transcriptome data [28,35]. The core of WGCNA is to group thousands of genes into modules based on their expression similarity. Genes within each module exhibit highly coordinated expression patterns and participate in common biological processes or regulatory pathways [35]. Through network topology analysis, hub genes within modules can be identified, which usually occupy central positions in the regulatory network and play important roles in module functions [36]. Therefore, WGCNA serves as an effective algorithmic complement to conventional transcriptome approaches that identify candidate genes based on differential expression [37]. The combination of transcriptome sequencing and WGCNA can capture the dynamic network structure of gene expression at a system-wide level and efficiently identify candidate functional genes associated with specific traits.

In the present study, we adopted the “starvation–feeding” nutritional intervention to amplify inherent differences in nutrition and metabolism among individuals, and combined RNA–seq with WGCNA to analyze the molecular basis of growth differences in *C. sonnerati*, identify key regulatory genes and pathways, aiming to address research gaps in this field, provide theoretical support for genetic improvement, and lay a foundation for precise breeding and sustainable aquaculture development.

## 2. Materials and Methods

### 2.1. Ethical Approval

The artificial challenge test carried out in this study was in accordance with the recommendations of the Care and Use of Laboratory Animals of the Chinese Academy of Fishery Sciences. The protocol of the test was approved by the Animal Care and Use Committee of the Chinese Academy of Fishery Sciences; the approval number is YSFRI–2025017.

### 2.2. Sample Collection

In this study, 8-month-old *C. sonnerati* were used as experimental animals, which were collected from Linlan Aquatic Co., Ltd., Wanning City, Hainan Province, China. Before the experiment, all fish were reared under standardized conditions in an indoor aquaculture workshop. Key water quality parameters were maintained as follows: water temperature of 23–26 °C, pH 7.8, salinity 30‰, and dissolved oxygen ≥ 5 mg/L. The fish were held in uniform concrete tanks measuring 10 m × 10 m and were fed a commercial diet twice daily to apparent satiation.

From this population, a total of 24 individuals with extreme body sizes (the largest and the smallest) were selected and divided into a large-size group and a small-size group, with 12 individuals in each group. Then, individuals in each group were randomly and equally divided into two subgroups, which were subjected to one-week starvation treatment or normal feeding treatment, respectively. Thus, a 2 (body size: big/small) × 2 (treatment: fasted/meal-fed) factorial experimental design was formed, resulting in four experimental groups with 6 fish per group. After the experiment, brain and muscle tissues of each fish were collected, immediately frozen in liquid nitrogen, and stored at −80 °C. For clear identification, the experimental groups were named by the combination of the initials of body size and treatment, defined as BF, BM, SF, and SM groups, respectively.

### 2.3. RNA Extraction, Library Construction, and Sequencing Analysis

Total RNA was extracted from tissues using TRIzol^®^ reagent (Invitrogen, Carlsbad, CA, USA). Subsequently, the concentration and purity of RNA were determined using a NanoDrop 2000 (Thermo Fisher Scientific, Waltham, MA, USA), with acceptable criteria as follows: the A260/A280 ratio ranged from 1.8 to 2.0, and A260/A230 > 2.0. The integrity of RNA was evaluated using an Agilent 2100 Bioanalyzer (Agilent Technologies, Santa Clara, CA, USA), with a qualified RNA Integrity Number (RIN) ≥ 7.0. For RNA samples that passed quality control, strand-specific transcriptome libraries were constructed using the Hieff NGS^®^ Ultima Dual-mode mRNA Library Prep Kit (Yeasen, Shanghai, China) [38]. Library construction was strictly performed in accordance with the kit instructions, and the main steps included the following: eukaryotic mRNA was enriched using oligo(dT) magnetic beads and random fragmentation was conducted; the first and second strands of cDNA were synthesized using fragmented mRNA as templates; end repair of double-stranded cDNA was performed, poly(A) tails were added, and sequencing adapters were ligated; after purification of ligation products using magnetic beads, PCR amplification with indexed primers was conducted to complete library enrichment. The constructed libraries were subjected to paired-end sequencing (150 bp) on the Illumina NovaSeq 6000 platform. To ensure the quality of analysis, raw reads were filtered for quality control using fastp software (v0.18.0) to remove low-quality reads and reads containing adapter sequences, yielding high-quality clean reads. HISAT2 (v2.2.1) software was used to align clean reads to the reference genome of *C. sonnerati* (NCBI accession number: GCA_043388425.1) [5,38]. Then, featureCounts was applied to calculate the raw read count of each gene based on alignment results. Finally, the read count data were normalized using the Transcripts Per Million (TPM) method.

### 2.4. Identification and Functional Enrichment Analysis of DEGs

Differential expression analysis was performed using the DESeq2 (v.1.42.0) R package [39]. This software assesses the significance (*p* value) of gene expression differences based on a negative binomial model, followed by correction using the False Discovery Rate (FDR). Genes with |log2FoldChange| > 0.585 (i.e., |Fold Change| > 1.5) and *p* < 0.05 were defined as significantly DEGs.

Gene Ontology (GO) functional enrichment and Kyoto Encyclopedia of Genes and Genomes (KEGG) pathway enrichment analyses were conducted for the identified significantly DEGs. For GO enrichment analysis, DEGs were mapped to the GO database. Using hypergeometric distribution tests, significantly enriched GO terms (*p* < 0.05) were screened in three categories—molecular function, cellular component, and biological process—relative to the whole-genome background, to reveal potential biological functions of the DEGs. KEGG pathway enrichment analysis was also based on hypergeometric distribution tests, aiming to systematically identify metabolic and signal transduction pathways significantly enriched by DEGs (*p* < 0.05), thereby illustrating their potential biological roles at the pathway level.

### 2.5. Weighted Gene Co-Expression Network Analysis (WGCNA)

Weighted gene co-expression network analysis (WGCNA) was employed in this study [35] to systematically analyze the co-expression patterns among genes and their associations with target traits. The analysis was performed using the BioNERO R package (v1.2.0) [40], following the specific workflow as below: First, the original gene expression matrix was rigorously filtered, including removing low-expression genes (median expression < 3 across all samples), performing ZK filtering based on co-expression patterns (Pearson correlation-based), and correcting principal components to eliminate potential batch effects, yielding high-quality expression data for subsequent analyses. The optimal soft threshold ensuring the network conformed to a scale-free topology (R^2^ > 0.8) was determined using the SFT_fit() function. A signed hybrid network was constructed using the exp2gcn() function with the following parameter settings: network type = “signed hybrid”, module merge threshold = 0.8, and minimum module size = 50. This function integrated steps including correlation calculation, topological overlap matrix (TOM) construction, hierarchical clustering, and dynamic tree cutting to automatically identify stable co-expression modules. After quality assessment of the obtained modules, the eigenvector values of each module were calculated. The Pearson correlation between modules and target traits was analyzed using the module_trait_cor() function to screen key modules significantly associated with the traits. Finally, core regulatory genes were identified based on the connectivity of genes within modules, and the gene interaction networks of important modules were imported into Cytoscape (v3.9.1) for visualization.

### 2.6. Quantitative Real-Time Polymerase Chain Reaction

Total RNA was extracted from brain and muscle tissue samples of *C. sonnerati* in each experimental group for quantitative real-time PCR (qRT–PCR) validation. Based on transcriptomic analysis results, four DEGs were randomly selected, and their qRT–PCR primers (sequences listed in Table 1) were designed using the PrimerQuest online tool (Integrated DNA Technologies, Coralville, IA, USA). First-strand cDNA was synthesized from 1 μg of total RNA using a commercial kit. qRT–PCR was performed on a LightCycler^®^ 480 system (Roche, Basel, Switzerland). The total reaction volume was 20 μL, containing 1 μL cDNA template, 0.6 μL forward and reverse primers each, 10 μL SYBR^®^ Premix Ex Taq™ II (Tli RNaseH Plus; TaKaRa, Tokyo, Japan), and 7.8 μL ddH_2_O. The reaction procedure was as follows: pre-denaturation at 95 °C for 5 min, followed by 35 amplification cycles (95 °C for 5 s, 56 °C for 30 s, 72 °C for 30 s), and finally melting curve analysis (65 °C to 95 °C at a heating rate of 0.1 °C/s) to confirm the specificity of amplified products. The *β-actin* gene was used as an endogenous control. The relative expression level of each gene was calculated using the 2^−ΔΔCt^ method, and the results were expressed as the mean ± standard error.

## 3. Results

### 3.1. RNA–Seq Analysis

Samples were collected from 8-month-old *C. sonnerati*, and their phenotypic data are shown in Table 2.

**Figure 1:**
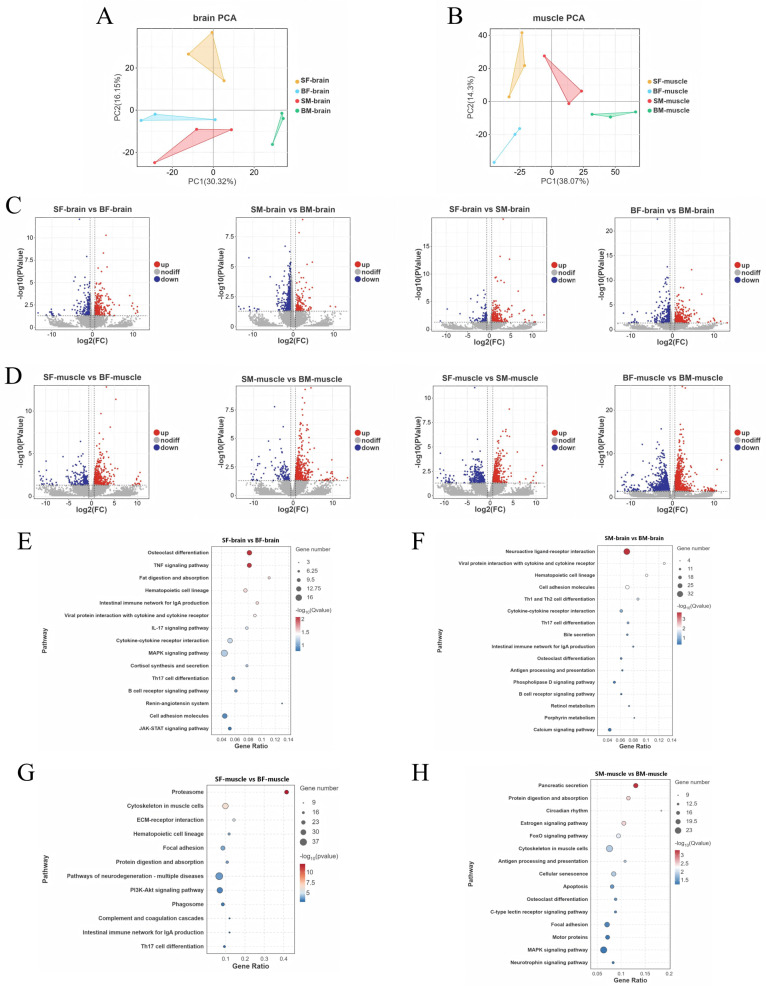
Comparative transcriptomic analysis of brain and muscle tissue samples. (**A**) Principal component analysis (PCA) plot showing the overall differences in gene expression profiles of brain tissue samples (SF, BF, SM, BM); (**B**) PCA plot showing the overall differences in gene expression profiles of muscle tissue samples (SF, BF, SM, BM); (**C**) volcano plots showing DEGs in different comparison groups of brain tissue (SF–BF, SM–BM, SF–SM, BF–BM); (**D**) volcano plots showing DEGs in different comparison groups of muscle tissue (SF–BF, SM–BM, SF–SM, BF–BM); (**E**) KEGG pathway enrichment analysis of the SF–BF comparison group in brain tissue; (**F**) KEGG pathway enrichment analysis of the SM–BM comparison group in brain tissue; (**G**) KEGG pathway enrichment analysis of the SF–BF comparison group in muscle tissue; (**H**) KEGG pathway enrichment analysis of the SM–BM comparison group in muscle tissue.

Transcriptome sequencing was performed on 24 tissue samples (brain and muscle) from different treatment groups. The raw reads of each sample ranged from 5.41 to 8.80 G. After quality control, high-quality clean reads ranged from 5.40 to 8.79 G. The Q20 and Q30 values of all samples were higher than 97% and 92%, respectively (Table A1). The RNA–seq data were deposited in the National Center for Biotechnology Information under accession number PRJNA1433042. Meanwhile, principal component analysis (PCA) showed that brain and muscle samples were clearly separated in the PCA plots (Figure 1A,B), indicating the reliability of the data for subsequent analysis.

DEGs among different treatment groups were analyzed. A total of 2553 DEGs were identified based on body size differences. In brain tissue, 471 DEGs (252 up-regulated, 219 down-regulated) were detected between small and large individuals under starvation (SF–brain vs. BF–brain). Under feeding conditions (SM–brain vs. BM–brain), 665 DEGs (120 up-regulated, 545 down-regulated) were found. In muscle tissue, 691 DEGs (464 up-regulated, 227 down-regulated) were identified under starvation (SF–muscle vs. BF–muscle), and 726 DEGs (596 up-regulated, 130 down-regulated) under feeding (SM–muscle vs. BM–muscle). Based on feeding treatment comparisons, 4480 DEGs were identified in total. In brain tissue, 492 DEGs (381 up-regulated, 111 down-regulated) were detected between starved and fed small individuals (SF–brain vs. SM–brain), and 905 DEGs (356 up-regulated, 549 down-regulated) between large individuals (BF–brain vs. BM–brain). In muscle tissue, 761 DEGs (455 up-regulated, 306 down-regulated) were found between treatments in small individuals (SF–muscle vs. SM–muscle), and 2322 DEGs (1299 up-regulated, 1023 down-regulated) in large individuals (BF–muscle vs. BM–muscle). The overall distribution of DEGs was visualized using volcano plots (Figure 1C,D).

To clarify the molecular mechanisms underlying body size differences in *C. sonnerati*, KEGG pathway enrichment analysis was performed on size-difference groups under the same feeding status in brain and muscle tissues. In brain tissue, growth was regulated through neuroendocrine and intracellular signaling. In the SF vs. BF group (Figure 1E), the “cell adhesion molecules” pathway related to cell communication and differentiation was enriched. In the SM vs. BM group (Figure 1F), “neuroactive ligand–receptor interaction”, which regulates neurotransmitter and hormone functions, showed the highest significance. The “calcium signaling pathway” was also enriched. These two pathways jointly regulate neuronal excitation and hormone release, which may systematically affect growth. In muscle tissue, pathways directly related to protein synthesis, metabolism, and muscle development were significantly enriched. In the SF vs. BF group (Figure 1G), DEGs were markedly enriched in the “proteasome” and “ECM–receptor interaction” pathways. The former regulates protein degradation, and the latter affects extracellular matrix signal transduction. Meanwhile, the “PI3K–Akt signaling pathway”, a core pathway controlling cell growth and survival, was also significantly enriched. In the SM vs. BM group (Figure 1H), DEGs were mainly enriched in “protein digestion and absorption” and the “FoxO signaling pathway”, which serves as a key hub linking nutrient sensing, metabolism, and growth regulation.

### 3.2. Identification of Core Overlapping Genes

**Figure 2:**
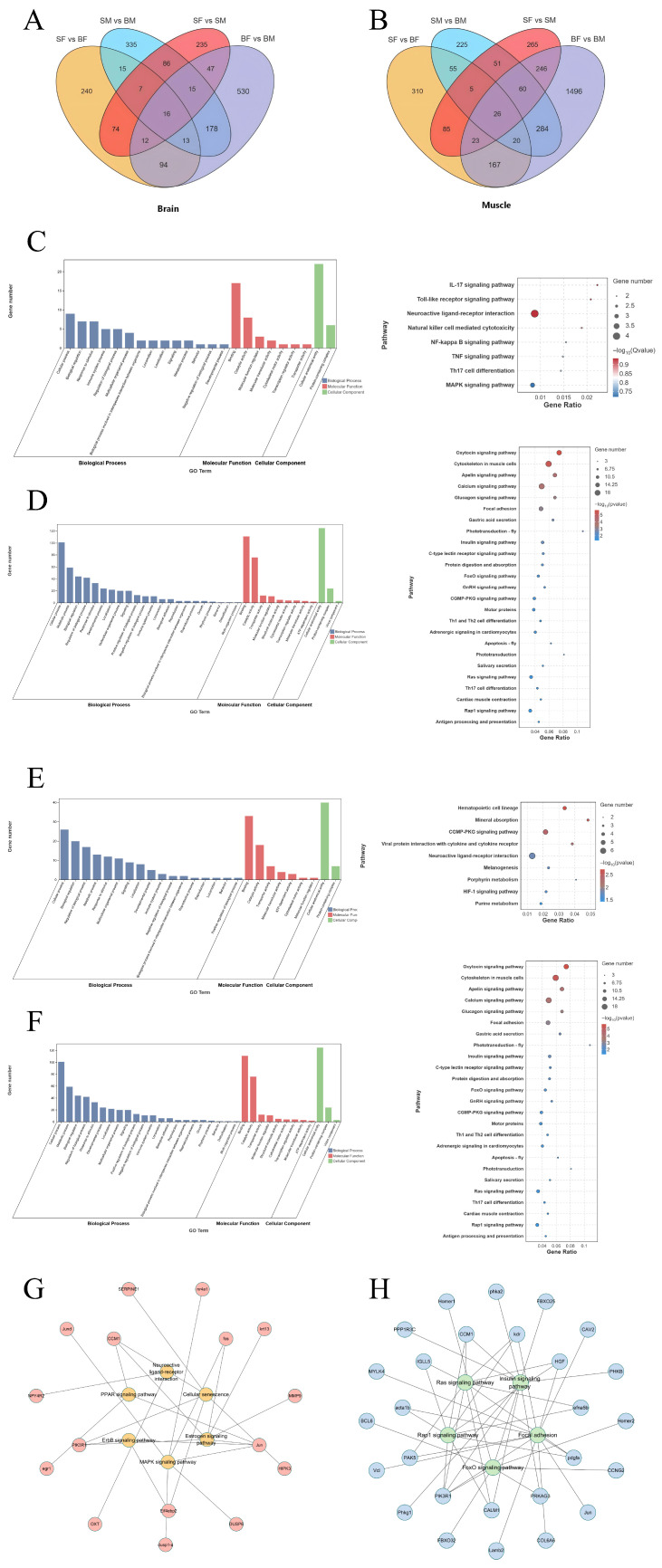
Screening, functional enrichment analysis, and regulatory network visualization of overlapping genes in brain and muscle tissues. (**A**) Venn diagram showing the overlap of DEGs among comparison groups (SF–BF, SM–BM, SF–SM, BF–BM) in brain tissue; (**B**) Venn diagram showing the overlap of DEGs among comparison groups (SF–BF, SM–BM, SF–SM, BF–BM) in muscle tissue; (**C**) GO functional enrichment analysis (left) and KEGG pathway enrichment analysis (right) of overlapping genes associated with body size differences in brain tissue; (**D**) GO functional enrichment analysis (left) and KEGG pathway enrichment analysis (right) of overlapping genes associated with body size differences in muscle tissue; (**E**) GO functional enrichment analysis (left) and KEGG pathway enrichment analysis (right) of overlapping genes associated with feeding differences in brain tissue; (**F**) GO functional enrichment analysis (left) and KEGG pathway enrichment analysis (right) of overlapping genes associated with feeding differences in muscle tissue; (**G**) regulatory network of pathways and core genes associated with growth/body size regulation; (**H**) regulatory network of pathways and core genes associated with feeding/metabolic regulation. All networks were constructed using Cytoscape software. Nodes represent genes or pathways, and edges represent gene–pathway affiliations.

To identify core candidate genes regulating body growth in *C. sonnerati*, Venn diagram analysis was performed to screen genes associated with body size differences under both feeding conditions. A total of 51 overlapping genes in brain tissue and 106 in muscle tissue were identified (Figure 2A,B). Subsequently, the biological functions of these genes were systematically analyzed using GO and KEGG pathway enrichment analyses (Figure 2C,D). Significantly enriched GO terms included “cellular process” (biological process), “catalytic activity” and “binding” (molecular function), and “cytosol” and “protein complex” (cellular component). Significantly enriched KEGG pathways included the “neuroactive ligand–receptor interaction” pathway, which plays a central role in neural signal transduction, as well as key metabolic and endocrine regulatory pathways such as the “estrogen signaling pathway” and the “Apelin signaling pathway”. These pathways are involved in multiple levels including neural signaling, hormone regulation, and cellular metabolism. These overlapping genes may lead to phenotypic changes by affecting these biological processes, forming a core network involved in growth regulation (Figure 2G).

To identify core genes responsive to feeding, Venn diagram analysis was also performed on individuals of the same body size under different feeding treatments, which screened 90 and 355 directly feeding-related genes in brain and muscle tissue, respectively (Figure 2A,B). GO and KEGG enrichment analyses of these genes were then conducted (Figure 2E,F). Significantly enriched GO terms included “cellular process” (biological process), “binding” (molecular function), and “cell part” and “cellular anatomical entity” (cellular component). The significantly enriched KEGG pathways included the “oxytocin signaling pathway”, the “Apelin signaling pathway”, and the “cytoskeleton in muscle cells” pathway. Together, these pathways constitute a core network involved in the regulation of growth and metabolic homeostasis (Figure 2H).

### 3.3. Weighted Gene Co-Expression Network Analysis (WGCNA)

To construct a gene co-expression network associated with growth and feeding in *C. sonnerati* and mine core genes, WGCNA was performed using an expression matrix containing 10,395 DEGs. With the change in soft threshold power, the optimal soft threshold β = 7 was determined according to the scale-free topology model fit (R^2^). The sample clustering dendrogram grouped samples based on gene expression similarity. A gene co-expression network associated with growth of *C. sonnerati* was constructed using the dynamic tree-cutting method, and 27 distinct co-expression modules were identified.

**Figure 3:**
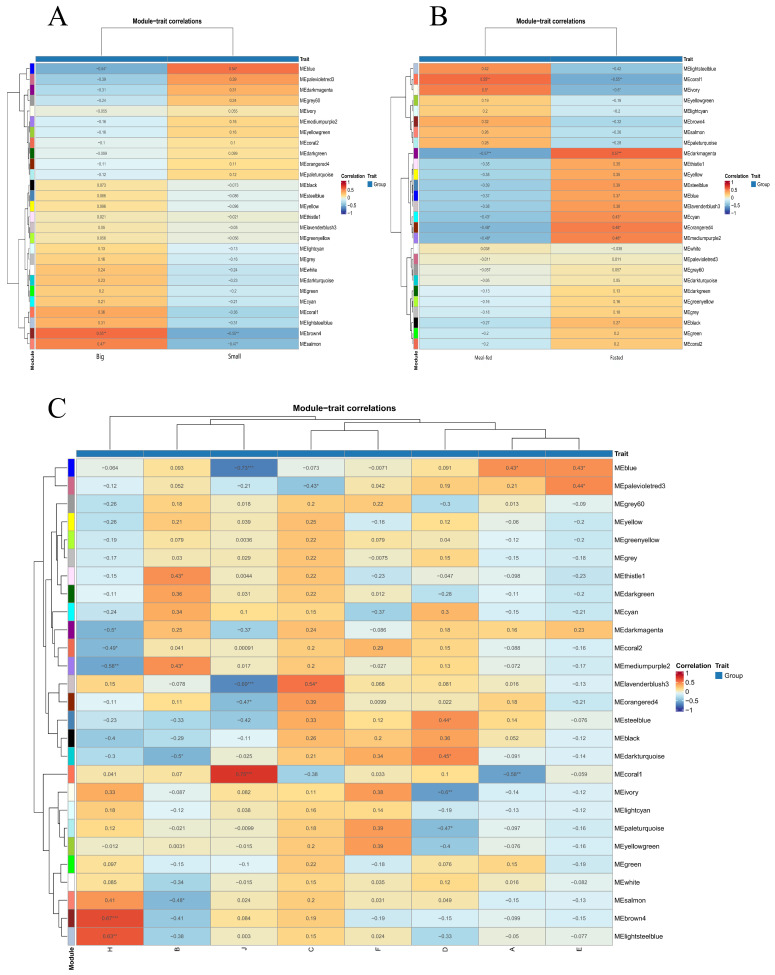
Module–trait correlations in the Weighted Gene Co–expression Network Analysis (WGCNA). (**A**) Heatmap of module correlations with the phenotypic traits big and small; (**B**) heatmap of module correlations with the phenotypic traits meal-fed and fasted; (**C**) heatmap of module correlations with the phenotypic traits A–H (A: SF–muscle, B: SF–brain, C: BF–muscle, D: BF–brain, E: SM–muscle, F: SM–brain, J: BM–muscle, H: BM–brain). In the heatmaps, the color of each cell indicates the magnitude and direction of the correlation coefficient, as defined by the color scale on the right (blue represents negative correlation; red represents positive correlation). Numbers inside the cells are the specific correlation coefficient values. Asterisks indicate statistical significance: * *p* < 0.05, ** *p* < 0.01, *** *p* < 0.001.

Module–trait correlation analysis revealed that several modules were significantly correlated with growth traits (Figure 3). The brown4 module showed a significant positive correlation with the “big” trait in the size group and a highly significant positive correlation with “H” (BM–brain) in the combined trait group. The blue module was significantly positively correlated with the “small” trait in the size group, showed significant positive correlations with “A” (SF–muscle) and “E” (SM–muscle), and exhibited a highly significant negative correlation with “J” (BM–muscle) in the combined trait group. The coral1 module demonstrated a significant positive correlation with “meal-fed” in the feeding trait group, and in the combined trait group, it was significantly positively correlated with “J” (BM–muscle) and significantly negatively correlated with “A” (SF–muscle). These modules are key for further exploration of the regulatory mechanisms governing growth and feeding in *C. sonnerati*.

### 3.4. Functional Enrichment and Regulatory Network Analysis of Module Genes

To elucidate the functions and regulatory networks of key modules, GO and KEGG enrichment analyses were performed for the brown4, blue, and coral1 modules identified by WGCNA. Gene interaction networks were constructed using Cytoscape.

**Figure 4:**
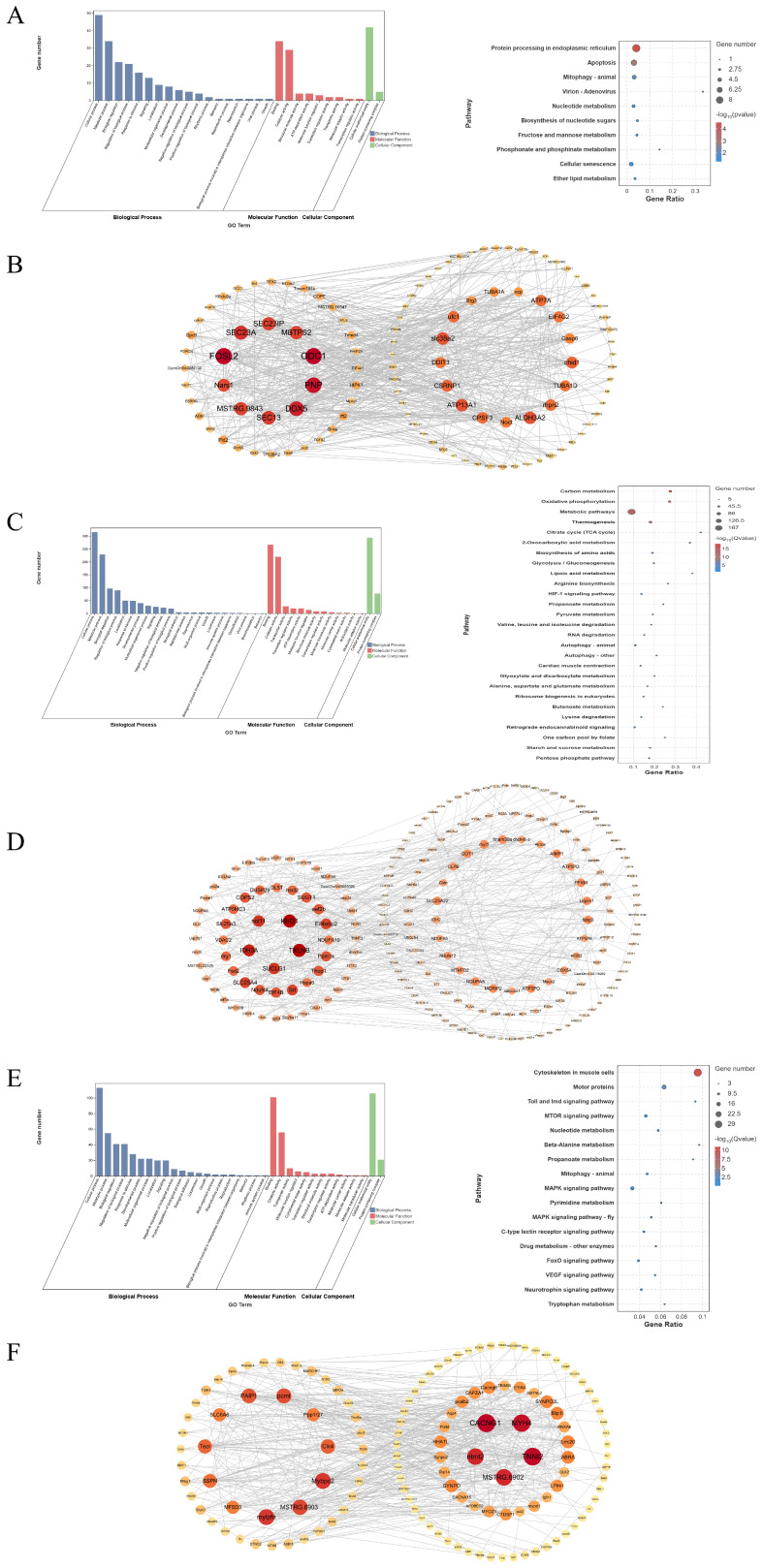
Functional enrichment analysis and gene interaction networks of key co-expression modules. (**A**) GO functional enrichment analysis and KEGG pathway enrichment analysis of genes in the brown4 module; (**B**) core gene network of the brown4 module; (**C**) GO functional enrichment analysis and KEGG pathway enrichment analysis of genes in the blue module; (**D**) core gene network of the blue module; (**E**) GO functional enrichment analysis and KEGG pathway enrichment analysis of genes in the coral1 module; (**F**) core gene network of the coral module.

GO enrichment analysis of the brown4 module (Figure 4A) indicated that the genes were primarily involved in “cellular process” and “macromolecule metabolic process” at the biological process level, enriched for “binding” at the molecular function level, and localized to the “cellular anatomical entity” at the cellular component level. KEGG pathway analysis (Figure 4B) revealed that the module genes were significantly enriched in anabolic pathways, including “fructose and mannose metabolism”, “pentose phosphate pathway”, and “glycerolipid metabolism”, as well as in homeostasis-related pathways such as “protein processing in endoplasmic reticulum” and “apoptosis”. Cytoscape network visualization (Figure 4C) demonstrated that the genes within the module formed a co-regulatory network centered on *eif2ak3*, *vcp*, *hspa8*, and *sec23a*.

GO enrichment analysis of the blue module (Figure 4D) indicated that the genes were primarily involved in “cellular process” at the biological process level, enriched for “binding” and “catalytic activity” at the molecular function level, and localized to the “protein-containing complex” at the cellular component level. KEGG pathway analysis (Figure 4E) revealed that genes in this module were significantly enriched in energy metabolism-related pathways, such as “carbon metabolism”, “oxidative phosphorylation”, “citrate cycle (TCA cycle)”, and “glycolysis/gluconeogenesis”, as well as in key signaling pathways including “insulin signaling pathway”, “AMPK signaling pathway”, and “HIF–1 signaling pathway”. Cytoscape network visualization (Figure 4F) showed that the genes formed an interaction network with *eif4ebp2*, *ppm1k*, *tsc1*, and *dusp29* as central hubs. This network also encompassed key genes directly involved in energy metabolism, such as aldoa, pfkm, and gys1.

GO enrichment analysis of the coral1 module (Figure 4) indicated that the genes were predominantly involved in “cellular process” and “metabolic process” at the biological process level, highly enriched for “binding” at the molecular function level, and localized to the “protein-containing complex” at the cellular component level. KEGG pathway analysis (Figure 4) revealed that module genes were significantly enriched in signal transduction pathways including MAPK, FoxO, and mTOR signaling pathways, metabolic regulatory pathways such as glycolysis, fatty acid degradation, and propanoate metabolism, and homeostasis maintenance pathways including autophagy and cellular senescence. Cytoscape network visualization (Figure 4) revealed that the genes formed an interaction network with *cacng1*, *pik3r1*, *map3k20*, and *dusp1–a* as core nodes.

### 3.5. Combined Analysis of RNA–Seq and WGCNA

**Figure 5:**
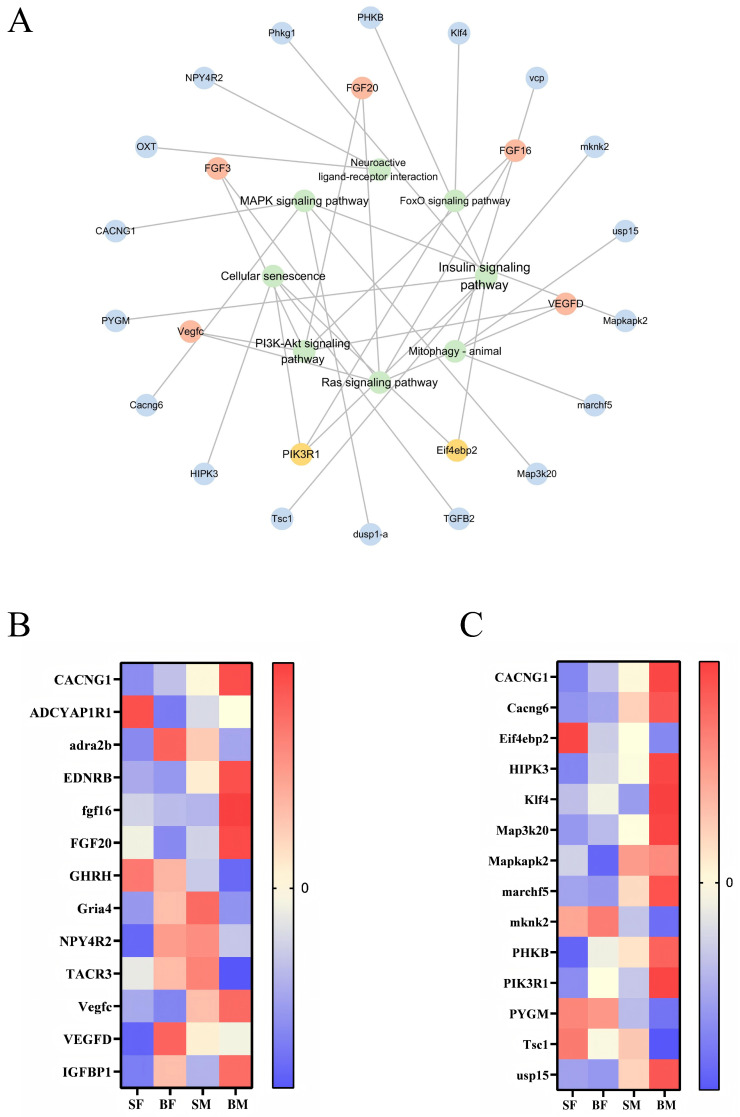
Construction of growth regulatory network model and validation of key gene expression. (**A**) Network of pathways and core genes related to growth regulation. The network was constructed using Cytoscape software, where nodes represent genes or pathways, and edges represent gene–pathway membership; (**B**) expression patterns of key regulatory genes in brain tissue; (**C**) expression patterns of key regulatory genes in muscle tissue. The standardized expression profiles (Z–score) of core genes in samples from each treatment group, with rows representing genes and columns representing sample groups.

Transcriptome differential expression analysis and WGCNA co-expression network analysis were integrated to systematically dissect the regulatory mechanisms underlying growth differences in *C. sonnerati*. First, core KEGG pathways closely related to growth were selected as the basic framework for constructing the regulatory network; these pathways were frequently identified from transcriptome analysis, overlapping genes, and module genes. Using hub genes from the three key modules (brown4, blue, and coral1) identified by WGCNA as the core, key functional molecules for the regulatory network were determined by intersection analysis between these genes and each pathway gene set. Combined with the differential expression patterns of these genes in brain and muscle tissues, a molecular network for growth regulation based on the “brain–muscle axis” synergistic effect was constructed (Figure 5A). This regulatory network systematically explains the molecular mechanisms underlying body size differences in *C. sonnerati* by integrating multiple biological processes, including neural signal sensing, intracellular signal transduction, muscle anabolism, and cellular homeostasis maintenance.

The gene expression heatmap of brain tissue (Figure 5B) revealed that large individuals (especially BM) showed a more active growth-promoting gene expression profile than small individuals. In large individuals, *ghrh* (related to neuroendocrine signal initiation), *npy4r2* (related to appetite regulation), and vascular endothelial growth factor family genes such as *vegfd*, *vegfc*, and *fgf20* were significantly highly expressed, while small individuals under starvation (SM) showed a potential growth-inhibiting pattern with high expression of *igfbp1*.

The gene expression heatmap of muscle tissue (Figure 5C) showed that multiple genes related to muscle growth and metabolism were significantly up-regulated in large individuals compared with small individuals. In large individuals, *pik3r1* (closely related to the insulin/PI3K–Akt signaling pathway), *tsc1* (negative regulator of mTORC1), and key glycogen metabolism enzyme genes *phkb* and *pygm* were significantly highly expressed, and the overall gene expression level was relatively low in small individuals.

### 3.6. qRT–PCR Validation

**Figure 6:**
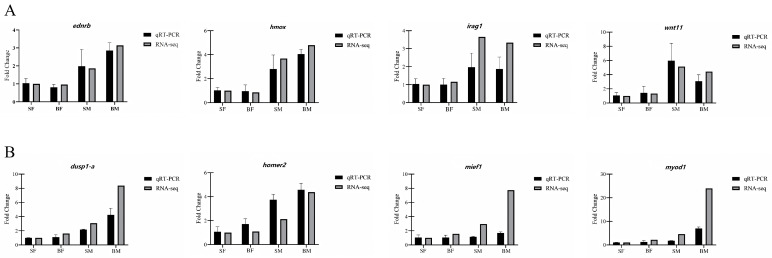
Multi-platform validation of key DEGs. (**A**) Expression validation of genes *ednrb*, *hmox*, *lrrc1*, and *wnt11* in brain tissue; (**B**) expression validation of genes *dusp1–a*, *homer2*, *mief1*, and *myod1* in muscle tissue. Expression data for each gene are presented as two sets of histograms: black bars represent the relative expression levels detected by qRT–PCR (normalized to the reference gene β-actin), and gray bars represent the Fold Change values obtained from RNA–seq. The vertical axis (*Y*-axis) indicates relative expression level (Fold Change); the horizontal axis represents the four experimental groups: SF, BF, SM, and BM.

To verify the reliability of the transcriptome sequencing results, total RNA was extracted from the brain and muscle tissues of *C. sonnerati* for qRT–PCR analysis. Four genes (*ednrb*, *hmox*, *irag1*, and *wnt11*) were randomly selected from brain tissue, and four genes (*dusp1–a*, *homer2*, *mief1*, and *myod1*) were randomly selected from muscle tissue. A total of eight DEGs were selected for validation. The expression change trends of genes obtained by qRT–PCR analysis were generally consistent with the RNA sequencing data (Figure 6A,B).

## 4. Discussion

### 4.1. Growth Integration in Brain Tissue: Synergy Between Neural Perception and Cellular Homeostasis

In the brain tissue of *Cephalopholis sonnerati*, growth-related DEGs include various regulatory factors. These factors consist of the initiator of the classical growth axis (*ghrh*), signal regulators (*igfbp1*), feeding center regulators (*npy4r2*), and growth-promoting and angiogenesis-related factors (*fgf16*, *fgf20*, *vegfd*). Functional enrichment analysis showed that growth regulation in brain tissue is mainly achieved through two core signaling pathways: the neuroactive ligand–receptor interaction pathway and the MAPK signaling pathway. The neuroactive ligand–receptor interaction pathway mediates neurotransmitter and neuropeptide signals. It regulates neuroendocrine loops such as the hypothalamic–pituitary axis, thereby coordinating growth and metabolism at the systemic level [41,42]. The MAPK signaling pathway is activated by growth factors such as FGFs and VEGFD. It transmits signals via the Ras–Raf–MEK–ERK kinase cascade, ultimately mediating intracellular responses including cell proliferation, differentiation, and survival [43,44].

The differential gene and pathway analyses in brain tissue were further supported by the brown4 co-expression module identified through WGCNA. Together, these results reveal the growth regulatory network of brain tissue. The brown4 module was significantly positively correlated with the “big” and “BM–brain” groups. KEGG enrichment analysis revealed that the module regulates growth through coordinated mechanisms at multiple levels. At the level of anabolism and energy supply, the module was significantly enriched in pathways such as fructose and mannose metabolism, pentose phosphate pathway, and glycerolipid metabolism. These pathways provide precursors and energy carriers for the synthesis of biological macromolecules, including proteins, nucleic acids, and lipids, thereby providing a material basis for tissue growth [45,46]. At the level of cellular quality control and homeostasis, the module was enriched in pathways such as protein processing, apoptosis, and cellular senescence. This indicates that, while supporting growth, the module also precisely regulates cell quantity and quality [47]. Genes in the module form a highly coordinated endoplasmic reticulum stress (ERS) network. Through stress sensing (kinase *eif2ak3*) and signal transduction (transmembrane protease *mbtps2* and pro-apoptotic transcription factor *ddit3*), the protein quality control system (retrotranslocation factor *vcp*, chaperone *hsc71*) processes misfolded proteins, ultimately guiding precise cell fate determination [48,49].

Growth regulation in the brain tissue of *C. sonnerati* is a highly integrated multi-level process. The neuroactive ligand–receptor interaction and MAPK signaling pathways receive and integrate growth and endocrine signals. These signals are then converted into specific cellular responses within the brown4 module. The central role of the MAPK pathway in fish growth regulation has been demonstrated in various fish species [50,51]. By synergistically enhancing anabolism and precisely regulating the ERS response, the module promotes tissue growth while strictly monitoring cell quality, thereby achieving a dynamic balance in brain tissue growth regulation.

### 4.2. Growth Regulation in Muscle Tissue: The Game Between Anabolism and Catabolism

In the muscle tissue of *C. sonnerati*, growth-related DEGs include several core molecules directly involved in growth regulation. These molecules consist of the negative feedback regulator of the MAPK signaling pathway (*dusp1*), the core subunit of the PI3K–Akt pathway (*pik3r1*), the protein translation inhibitor (*eif4ebp2*), and the rate-limiting enzyme of glycolysis (*pfkm*). Functional enrichment analysis showed that the growth regulation in muscle tissue is achieved through the synergy of multiple levels and signaling pathways. At the level of signal transduction, ligand–receptor binding in the oxytocin signaling pathway activates the downstream MAPK signaling pathway and calmodulin-dependent kinases, thereby affecting muscle protein synthesis [52]. At the level of energy metabolism and anabolism, the insulin signaling pathway activates the PI3K–Akt–mTORC1. It promotes protein translation, glucose uptake and glycogen synthesis, thereby providing the material and energy basis for muscle growth [53,54]. The antagonistic FoxO signaling pathway is activated when nutrients or energy are limited. By up-regulating the expression of related genes, it promotes protein degradation and autophagy, maintaining metabolic homeostasis and limiting growth [55].

WGCNA co-expression network analysis revealed the dynamic balance of growth in muscle tissue at the system level. The coral1 module was significantly positively correlated with the BM–muscle group and significantly negatively correlated with the SF–muscle group. Its gene enrichment results construct a network that drives anabolism and tissue growth. Genes in the module are involved in the signaling network that drives cell proliferation and differentiation, enriched in key pathways such as MAPK, FoxO, and mTOR, thereby responding to external stimuli and regulating growth. The functional network is supported by corresponding hub genes: *pik3r1*, as a key regulatory subunit of the insulin/PI3K–Akt pathway, integrates growth and metabolic signals; kinase genes *map3k20* and *mapkapk2*, together with transcription factor *klf4*, form a signaling regulatory network. They are widely involved in the feedback regulation of insulin, growth factor, and other pathways, as well as RNA processing [56,57,58]. Through synergistic effects, genes and pathways direct energy to anabolism and cell proliferation, thereby prioritizing tissue growth and development.

In contrast, the blue module was significantly positively correlated with the SF–muscle and SM–muscle groups. Its gene function enrichment indicates a clear “growth restriction and homeostasis maintenance” network. Genes in the module are widely involved in signal transduction and metabolic regulation processes: at the level of signal transduction, the module is enriched in insulin and AMPK pathways, regulating growth by sensing energy status; at the level of metabolic regulation, genes in the module are significantly enriched in pathways such as glycolysis, citrate cycle, oxidative phosphorylation, and amino acid and fatty acid metabolism, synergistically maintaining basic energy supply and biomolecule synthesis. Hub genes of the blue module form a coordinated inhibitory network, including *eif4ebp2* (inhibiting translation initiation through mTOR signaling), *ppm1k* (activating AMPK-related metabolic regulation), *tsc1* (negatively regulating mTORC1 signaling), and genes involved in energy metabolism: *aldoa* and *pfkm* (regulating glycolysis), as well as *gys1* and *pygm* (regulating glycogen synthesis and decomposition) [53,59]. Hub genes show high overlap with DEGs in the transcriptome. This suggests that under limited conditions, individuals can actively inhibit anabolism and growth, allocate limited resources to maintain basic life activities, and prioritize survival homeostasis at the cost of growth.

Growth of *C. sonnerati* muscle tissue is coordinately regulated by multiple pathways. Growth-promoting pathways such as insulin/PI3K–Akt–mTOR and MAPK drive anabolism, while pathways such as AMPK and FoxO limit growth to maintain homeostasis. The insulin/PI3K–Akt cascade pathway and FoxO signaling pathway are also enriched in fast- and slow-growing individuals of *Megalobrama amblycephala*, indicating that these pathways are a universal regulatory method in fish growth [60]. WGCNA confirmed this result at the system level: the coral1 module is enriched in genes driving anabolism and cell proliferation, directly promoting protein synthesis and energy metabolism; the blue module is enriched in genes maintaining metabolic homeostasis and limiting growth, accurately inhibiting anabolism and activating decomposition processes. Muscle growth is ultimately determined by achieving a dynamic balance between anabolic processes that promote growth and catabolic processes that prioritize survival and maintenance [61].

### 4.3. Cascade Amplification Effect of Feeding: Systemic Enhancement of Growth Pathways

Among the DEGs induced by feeding in *C. sonnerati*, several core molecules are directly involved in metabolism and growth regulation. These molecules include the key regulatory subunit of the insulin/PI3K–Akt signaling pathway (*pik3r1*), phosphorylation regulatory enzymes involved in glycogen metabolism (*phkb*, *phkg1*), and the cytoskeletal protein (*vcl*), which integrates external signals with intracellular structures. These genes constitute key nodes in signal transduction and metabolic reprogramming. Functional enrichment analysis showed that feeding-induced gene changes were significantly enriched in signaling pathways such as insulin, Wnt, Ras, Rap1, and FoxO, forming a coordinated regulatory network. Within this network, the insulin and Wnt signaling pathways are located upstream and are responsible for initiating growth signals and promoting anabolism and cell fate determination, respectively [62]. The Ras and Rap1 signaling pathways receive and integrate these signals and, by activating downstream pathways such as MAPK and PI3K–Akt, convert them into specific cell cycle processes and gene expression changes [63,64]. The FoxO signaling pathway plays a key role in negative feedback regulation, forming a bidirectional regulatory relationship with other pathways and jointly maintaining the balance between metabolism and growth [60,65].

The feeding-activated signaling network was further supported by the coral1 co-expression module identified through WGCNA. The coral1 module was significantly positively correlated with the “meal-fed” group. Genes in this module convert nutritional and metabolic signals into growth-related outputs through a coordinated network. At the level of metabolic regulation, pathways such as glycolysis and fatty acid degradation, which are enriched in this module, directly provide energy and biosynthetic precursors for cell growth, while their metabolites also act as key regulatory signals. These metabolic signals are accurately sensed by pathways such as mTOR and AMPK. Specifically, mTOR, as a central nutrient sensor, is positively regulated by glucose and amino acid levels, whereas AMPK, as an energy sensor, is activated under low-energy conditions [66,67]. At the level of signal transduction, these metabolic states regulate mTOR, AMPK, and their downstream pathways, including FoxO and MAPK, thereby determining the balance between cellular anabolism and catabolism and ultimately regulating cell proliferation, differentiation, and growth [68,69]. Hub genes within this module form a coordinated growth-promoting network. At the metabolic and execution levels, *phkb* and *phkg1* (involved in glycogen metabolism and energy storage), *slc6a6* and *mfsd3* (responsible for taurine and substrate transport), and *paip1* (promoting protein translation) jointly support cellular energy supply and material conversion. Growth regulation via metabolic pathways has been widely reported in fish species [23,61,70], indicating that under nutrient-sufficient conditions, individuals can actively promote cell proliferation and tissue development through the coordinated enhancement of signal transduction, metabolic activity, and biosynthetic capacity.

Feeding activates a growth-promoting signaling network in *C. sonnerati*, in which the insulin and Wnt pathways initiate growth signals, while the Ras and Rap1 pathways mediate signal integration and transduction and enhance the activity of downstream pathways such as MAPK and PI3K–Akt. Meanwhile, the FoxO pathway plays a key role in negative feedback regulation, jointly maintaining growth and metabolic homeostasis. WGCNA further showed that the coral1 module is enriched in similar pathways, forming a coordinated process from metabolic support to signal-driven regulation at the transcriptome level. Notably, the role of glycolysis-related pathways in growth regulation through metabolic control has been demonstrated in various fish species, including *Megalobrama amblycephala*, *Scophthalmus maximus,* and *Spinibarbus denticulatus* [23,60,66]. These findings indicate that feeding converts nutrient input into signals that drive tissue growth by systematically enhancing the activity of multi-level regulatory pathways [26,27].

### 4.4. Brain–Muscle Coordinated Regulation: From Systemic Perception to Tissue Execution

By integrating transcriptome and WGCNA, this study systematically revealed the molecular mechanisms underlying body size differences in *Cephalopholis sonnerati*. Based on the coordinated effects of the brain, muscle, and feeding status, a multi-level and multi-pathway coordinated “brain–muscle” growth regulatory model was constructed. The complete regulatory chain from “signal perception–cascade transmission–tissue execution–homeostasis maintenance” was clarified, providing a theoretical framework for understanding growth regulation in *C. sonnerati*.

As the “signal center” of growth regulation, brain tissue undertakes the core functions of initial signal perception and initiation. This study identified multiple growth-related DEGs, including *igfbp1*, *fgf16*, and *npy4r2*, in individuals with extreme body sizes. Among these, genes such as *npy4r2* and *oxt* were significantly enriched in the neuroactive ligand–receptor interaction pathway. Serving as a key interface for responding to environmental and nutritional signals, this pathway accurately perceives signals such as neurotransmitters and growth factors, and converts them into transmittable biological responses, constituting the initial step in growth regulation [41,42]. Meanwhile, this aligns with the feeding-induced signal initiation, where nutritional signals amplify growth regulation through this pathway. Notably, similar neuroendocrine mechanisms have been widely reported in teleost fish, suggesting a conserved role, although its prominence here may reflect species-specific adaptations. The initial growth signals are transmitted across tissues via the Ras signaling pathway. This pathway is significantly enriched in both brain and muscle tissues across individuals of different sizes, consistent with its role as “a signal hub.” Activated Ras protein acts as a key molecular switch, triggering downstream programs and activating the MAPK and PI3K–Akt pathways to precisely regulate cell proliferation, differentiation, and survival [64,71]. Key components of the MAPK pathway include calcium channel regulatory genes (*cacng1*, *cacng6*), phosphatase *dusp1–a*, and kinases (*map3k20*, *mapkapk2*). These genes can jointly transmit rapid instructions for cell proliferation and differentiation [58]. Muscle tissue serves as the “execution terminal” of growth regulation, receiving growth signals from brain tissue and completing specific growth processes. In muscle tissue, the activated PI3K–Akt pathway, via its core regulatory subunit *pik3r1*, collaborates with insulin pathway-related genes—including translation regulators (*eif4ebp2*, *mknk2*), glycogen metabolism-related genes (*phkb*, *phkg1*), and upstream inhibitor *tsc1*—to directly regulate processes such as glucose uptake, glycogen synthesis, and protein translation, thereby providing the material and energy basis for muscle mass maintenance and increase [61,72]. Additionally, PI3K–Akt phosphorylates FoxO proteins, retaining them in the cytoplasm and inhibiting their transcriptional activity. This removes inhibitory programs mediated by FoxO, such as cell cycle arrest and autophagy activation, thereby facilitating anabolic metabolism and cell growth [73]. This mechanism maintains the balance between anabolism and catabolism in muscle, dynamically regulating muscle growth. The dynamic balance of growth and metabolic signals ultimately leads to two critical terminal biological processes, cellular senescence and mitophagy, ensuring the healthy growth of *C. sonnerati*. The cellular senescence pathway integrates genes such as the translation inhibitor (*eif4ebp2*), growth signal element (*pik3r1*), and cytokine (*tgfb2*) to precisely regulate growth and homeostasis. The mitophagy pathway performs organelle quality control through ubiquitination-related genes (*marchf5*, *usp15*) and the retrograde transport factor (*vcp*) [68]. These processes coordinate with muscle metabolic homeostasis and feeding-induced energy reprogramming, collectively maintaining cell balance and organismal health during rapid growth.

Functional analysis of hub genes provides direct molecular evidence for the “brain–muscle” growth model, and these genes were consistently identified in both differential expression analysis and WGCNA. Among them, *pik3r1* (encoding the regulatory subunit p85α of PI3K) acts as a core hub, responsible for integrating and transducing upstream growth signals from brain tissue and feeding-induced nutritional signals [56]. As a key subunit of the PI3K complex, p85α inhibits the activity of the catalytic subunit p110 in the resting state. Upon transmission of insulin or IGF signals, its SH2 domain binds phosphorylated receptor tyrosine kinases, relieving autoinhibition and recruiting PI3K to the membrane, initiating Akt–mTOR signaling [74]. The expression and functional states of this gene directly determine the signal intensity of the PI3K–Akt–mTOR growth pathway, further regulating cell proliferation, metabolic adaptation, and tissue growth [75]. Functional validation in teleosts like gibel carp (*Carassius gibelio*) shows that heterozygous *pik3r1* depletion leads to p85α haploinsufficiency, paradoxically enhancing PI3K–AKT–mTOR activity. Mutant fish show enhanced somatic growth and feed conversion, highlighting their potential application in aquaculture through pathway activation [76]. Another core hub gene, *eif4ebp2* (encoding 4E–BP1), is a downstream effector of the mTORC1 signaling pathway, responsible for converting growth signals into translation initiation instructions [77]. Non-phosphorylated 4E–BP1 inhibits translation initiation by binding eIF4E. When upstream growth signals are activated through the PI3K–Akt–mTORC1 axis, mTORC1 phosphorylates 4E–BP1, altering its conformation and relieving the inhibition on eIF4E. This initiates efficient mRNA translation, synthesizes key proteins required for the cell cycle and metabolism, and directly converts growth signals into protein synthesis capacity that drives cell volume increase and tissue growth. Its role as a nutrient and energy status sensor is highlighted in teleost studies. For instance, in mandarin fish (*Siniperca chuatsi*), *eif4ebp2* expression is up-regulated under fasting conditions, switching from growth promotion to growth inhibition and metabolic maintenance. This indicates that *eif4ebp2* integrates GH/IGF and mTOR signals to balance growth and metabolism under varying nutrition [78].

In summary, the growth regulation of *C. sonnerati* is a systematic process involving “brain–muscle” coordination, multi-pathway interaction, and multi-gene synergy. Brain tissue, as the signal center, perceives signals and initiates growth programs through the neuroactive ligand–receptor interaction pathway. The Ras pathway, acting as a signal hub, transmits upstream signals to the two core pathways, MAPK and PI3K–Akt. Muscle tissue, as the execution terminal, regulates the balance between anabolism and catabolism through pathways such as PI3K–Akt to complete growth execution. The cellular senescence and mitophagy pathways maintain growth homeostasis. Hub genes like *pik3r1* and *eif4ebp2* serve as regulatory nodes to integrate internal and external signals, achieving precise regulation of growth rate and extent. This coordinated regulatory model integrates all core results in the Section 4 of this study, clearly revealing the molecular mechanisms underlying body size differences in *C. sonnerati*, and providing important molecular targets and theoretical support for the genetic improvement of growth traits and the optimization of aquaculture in *C. sonnerati*. From an applied perspective, these findings provide valuable insights for aquaculture. The identified hub genes, such as *pik3r1* and *eif4ebp2*, represent promising candidate markers for genomic selection aimed at improving growth traits. In addition, key pathways such as PI3K–Akt–mTOR offer potential targets for nutritional intervention, including the development of functional feeds that enhance anabolic metabolism and optimize energy utilization. These strategies provide a clear translational pathway from molecular mechanisms to practical applications in aquaculture breeding and management. This study is based on transcriptomic data, which may not fully reflect protein-level regulation, particularly for pathways such as PI3K–Akt and MAPK that are largely controlled by post-translational modifications. Future studies integrating proteomics, phosphoproteomics, and functional validation will be essential to achieve a more comprehensive understanding of growth regulation.

## 5. Conclusions

By integrating transcriptome and WGCNA, this study constructed a cascade regulatory network for growth differences in *Cephalopholis sonnerati*, revealing the core mechanism from signal perception in brain tissue (e.g., neuroactive ligand–receptor pathway) to metabolic execution in muscle tissue (via the Ras–MAPK/PI3K–Akt axis). Key hub genes *pik3r1* and *eif4ebp2* act as “regulatory knobs” in the network, integrating nutritional signals and precisely regulating downstream growth programs. This study clarifies the molecular basis of body size differences in *C. sonnerati*, provides new insights into the regulatory mechanisms of fish growth, and offers a basic theoretical basis and candidate genes for the genetic improvement of growth traits in *C. sonnerati*.

## Figures and Tables

**Table 1:** Specific primers for qRT–PCR.

Primer Type	Primer Sequences
*ednrb* F	TGGTGGTTGTTTGGCTTCTATTT
*ednrb* R	CTACTTCCCTCCGCTGTTTCAT
*hmox* F	GAGTCACCCTGCCGCAGTAC
*hmox* R	GTTCAGTCGGGAAGTAAATGGGT
*irag1* F	TCGTCCTCACCGTGTTCATCTA
*irag1* R	AGGGCTTCCAGTCCACCAA
*wnt11* F	ACTTCTGCGATAAGAACGACAAAC
*wnt11* R	CACTGCCGAGGGAGGTTTT
*dusp1–a* F	CGGTTAGGAATAAAGGAGGTCG
*dusp1–a* R	GGTGGCGGAGCGGGAG
*homer2* F	CGCAACAGCTACCGCATCAT
*homer2* R	GAAGCGAAGCCCAGTCCAA
*mief1* F	CTGTCTAATGCTCGGCTGGTT
*mief1* R	AGCCATCTTCTGCTCCACCTT
*myod1* F	GGAAAGGCGACGGCTCG
*myod1* R	TTACTGCTGCTGGAATCGTCTG
*β–actin* F	TGGCATCACACCTTCTACAATGAG
*β–actin* R	TCACACCATCACCAGAGTCCAT

**Table 2:** Morphometric indices of experimental fish.

Group	Total Length/cm	Body Depth/cm	Body Weight/g
BM	16.9	6.4	125
BM	18.6	7.4	149
BM	16	6.4	119
BM	17.5	6.3	128
BM	17.2	6.2	131
BM	17.9	6.6	143
SM	13.7	4.5	51
SM	15.2	4.9	70
SM	14.6	5.1	71
SM	13.7	4.4	55
SM	11.6	3.8	32
SM	12.3	4.2	41
BF	18.5	7	140
BF	18.2	7.8	160
BF	18.8	7.2	165
BF	17.2	6.3	125
BF	17.1	6.6	106
BF	15.5	5.7	89
SF	14.6	4.8	61
SF	13.6	4.7	49
SF	13.2	4.1	49
SF	14.7	4.9	67
SF	14.5	5	71
SF	12.6	4.3	41

## Data Availability

The RNA–seq data were deposited in the National Center for Biotechnology Information under accession number PRJNA1433042.

## Abbreviations

The following abbreviations are used in this manuscript:

SF	Small Fasted
BF	Big Fasted
SM	Small Meal-fed
BM	Big Meal-fed

## Appendix A

**Table A1:** Sequencing data quality status.

Sample	Raw Reads	Clean Reads	Q20 (%)	Q30 (%)	GC (%)
SF–muscle1	5,411,855,700	5,404,370,499	97.43%	93.17%	51.65%
SF–muscle2	6,119,871,000	6,107,941,866	97.80%	94.02%	51.37%
SF–muscle4	5,624,858,400	5,617,103,140	97.16%	92.58%	51.29%
SF–brain1	7,634,809,500	7,598,159,881	98.24%	95.32%	46.19%
SF–brain2	7,537,353,600	7,524,792,459	97.59%	93.51%	45.56%
SF–brain4	7,729,600,500	7,721,182,039	97.44%	93.21%	46.19%
BF–muscle2	5,872,833,000	5,858,135,335	97.50%	93.40%	51.61%
BF–muscle3	6,221,660,700	6,197,374,320	97.44%	93.00%	51.50%
BF–muscle4	6,128,278,800	6,112,391,982	97.57%	93.60%	51.44%
BF–brain2	7,239,044,100	7,225,635,825	97.75%	93.98%	45.82%
BF–brain3	6,782,950,800	6,770,010,918	97.25%	92.74%	46.56%
BF–brain4	7,787,181,600	7,775,003,521	97.35%	92.98%	45.83%
SM–muscle1	6,959,808,000	6,946,457,800	97.39%	93.09%	51.73%
SM–muscle2	7,013,672,700	7,003,037,542	97.54%	93.48%	51.39%
SM–muscle3	6,200,519,100	6,187,763,552	97.44%	93.20%	51.79%
SM–brain1	8,803,503,900	8,790,542,552	97.40%	93.08%	46.17%
SM–brain2	6,892,567,200	6,878,539,769	97.47%	93.27%	46.32%
SM–brain3	7,889,676,900	7,874,675,472	97.60%	93.62%	46.21%
BM–muscle1	6,861,443,400	6,851,902,848	97.34%	92.95%	51.28%
BM–muscle2	7,593,715,800	7,581,396,681	97.15%	92.60%	51.66%
BM–muscle3	7,224,552,900	7,213,951,424	97.42%	93.13%	51.30%
BM–brain1	5,950,234,500	5,943,027,696	97.09%	92.32%	46.39%
BM–brain2	6,871,426,800	6,861,387,357	97.87%	94.01%	46.42%
BM–brain3	6,763,189,200	6,752,102,131	97.39%	93.07%	46.50%

**Table A2:** Summary of pathway enrichment analysis.

Pathway	Module	Overlapping Genes	DEGs (Transcriptome)
Neuroactive ligand–receptor interaction		Brain–big + small Brain–meal-fed + fasted	BrainSM-BM
MAPK signaling pathway	Coral1	Muscle–big + small	Brain SF-BF Muscle SM-BM
Ras signaling pathway	Blue	Muscle–meal-fed + fasted	Brain SM-BM Muscle SM-BM
PI3K-Akt signaling pathway	Blue Coral1	Muscle–big + small	Muscle SF-BF Muscle SM-BM
Insulin signaling pathway	Blue	Muscle–meal-fed + fasted	Muscle BF-BM
FoxO signaling pathway	Coral1	Muscle–meal-fed + fasted	Muscle SM-BM
Cellular senescence	Brown	Muscle–big + small	Muscle SM-BM
Autophagy–animal	Brown Coral1		Muscle BF-BM

**Table A3:** Summary of identified gene sets.

Gene	Pathway	Module
*cacng1*	MAPK signaling pathway	Coral1
*cacng6*	MAPK signaling pathway	Coral1
*dusp1-a*	MAPK signaling pathway	Coral1
*map3k20*	MAPK signaling pathway	Coral1
*mapkapk2*	MAPK signaling pathway	Coral1
*klf4*	FoxO signaling pathway	Coral1
*marchf5*	Mitophagy–animal	Coral1
*usp15*	Mitophagy–animal	Coral1
*vcp*	Mitophagy–animal	Brown
*hipk3*	Cellular senescence	Coral1
*tgfb2*	Cellular senescence	Brown
*mknk2*	Insulin signaling pathway	Blue
*phkb*	Insulin signaling pathway	Coral1
*phkg1*	Insulin signaling pathway	Coral1
*pygm*	Insulin signaling pathway	Blue
*tsc1*	Insulin signaling pathway	Blue
*pik3r1*	FoxO signaling pathway Insulin signaling pathway Cellular senescence	Coral1
*eif4ebp2*	Cellular senescence Insulin signaling pathway	Blue
